# Does perceived stigma impact opioid use disorder treatment uptake? A cross-sectional secondary analysis of buprenorphine engagement among syringe service program participants

**DOI:** 10.1186/s13722-026-00665-3

**Published:** 2026-04-10

**Authors:** Bailey Campbell, Lindsey R. Riback, Juan Gatica Portillo, Stephanie Maricic, Aaron D. Fox

**Affiliations:** 1 Department of Medicine, Albert Einstein College of Medicine, 1300 Morris Park Avenue, Bronx, NY 10461 USA; 2 Division of General Internal Medicine, Department of Medicine, Montefiore Medical Center, 3300 Kossuth Avenue, Bronx, NY 10467 USA

## Abstract

**Introduction:**

Buprenorphine is an effective medication for opioid use disorder (OUD) treatment; however, stigma surrounding substance use may deter individuals from seeking treatment and contribute to lower engagement once treatment is initiated. This study examined relationships between substance use stigma and buprenorphine treatment engagement among syringe service program (SSP) participants.

**Methods:**

This cross-sectional secondary analysis used data from a randomized control trial of buprenorphine treatment initiation at three SSPs. Participants met DSM-5 criteria for OUD and expressed interest in buprenorphine. Participants completed the Ahern substance use stigma scale at baseline. The primary outcome was buprenorphine engagement, defined as having an active prescription 30 days post-enrollment. Multivariable logistic regression examined associations between participants’ perceived stigma and buprenorphine engagement.

**Results:**

Ninety-seven participants with a mean age of 42 years reported high levels of substance use stigma across multiple domains (discrimination, alienation, perceived devaluation); however, there were no significant differences in stigma scores between those who were and were not engaged in buprenorphine at 30 days. Participants who reported being denied medical care previously due to their substance use were significantly more likely to engage in buprenorphine (adjusted Odds Ratio = 2.64, 95% CI = 1.04–6.66).

**Discussion:**

Stigma scores did not predict buprenorphine engagement, but unexpectedly, participants who had experienced discrimination in medical care were more likely to engage. While stigma was not associated with buprenorphine uptake, most participants reported high levels of stigma, highlighting the need for patient-centered models of care that mitigate stigma.

## Introduction

Buprenorphine is a safe and effective opioid use disorder (OUD) treatment, yet adoption has been sluggish, even during a more than two decade-long opioid overdose epidemic [1]. Buprenorphine reduces opioid cravings, non-prescribed opioid use, opioid overdoses and opioid-related morbidity [2–4]. Despite these benefits, both systemic and individual barriers prevent people with OUD from starting buprenorphine treatment. Costs, health insurance coverage, provider availability (particularly in rural or underserved areas), and stigma towards people who use drugs are well-described barriers to buprenorphine treatment [5]; however, stigma can operate in different ways and deserves greater attention for its role in treatment seeking behaviors.

Stigma encompasses societal disapproval and marginalization of individuals with specific identities or behaviors, including substance use, which results in discriminatory treatment and diminished societal status [6]. Stigma operates through labeling, stereotyping, separation, and status loss within a particular framework of power, meaning that stigma processes serve to disempower the stigmatized groups [6]. Stigma functions as a social mechanism employed by dominant groups to impose conformity or exclude stigmatized groups, thereby creating psychological and social challenges that the stigmatized must navigate during everyday life [7]. For stigmatized individuals, these circumstances can foster negative self-perceptions, which degrades their self-esteem and self-efficacy, reinforcing the stereotypes imposed by the larger society, and ultimately furthering their isolation [6, 8].

Stigma can hinder individuals with substance use disorders (SUDs) from engaging in treatment by creating social, psychological, and structural barriers [9]. From a structural perspective, the healthcare system in the United States reinforces stigma. Healthcare providers often lack training to identify and manage SUDs, practice within systems where SUD treatment has been historically separated from other medical care, use stigmatized terminology that influences their behaviors, and may harbor negative attitudes toward patients with SUDs [10–13]. For people who inject drugs (PWID), stigma often leads to delays in seeking care or avoiding disclosure of their substance use history to healthcare providers [14–18]. In qualitative studies, PWID express fear of being mistreated or discriminated against by healthcare providers, which reflects the impact of societal stigma [16–18]. People with SUDs also have high levels of self-stigma or internalized stigma, meaning they believe that societal labels and stereotypes apply to themselves as individuals [19–21]. This process of self-stigmatization has been linked to impaired social functioning including social withdrawal and decreased engagement in interpersonal activities, including seeking SUD treatment [20]. Self-stigma has a greater effect on the quality of life of those using opioids than those who use alcohol [22]. Other studies show that individuals with OUD seeking inpatient detoxification face ongoing challenges with stigma, which may deter them from returning to treatment when needed after previous attempts despite the potential benefits [23].

When applied to buprenorphine treatment, stigma may function through social policies and individual behaviors. Compared with other medical services, the greater regulatory oversight and restrictions on buprenorphine reinforces the stigma that medications for addiction treatment “substitute one addiction for another,” and therefore must be contained instead of expanded [5, 24]. Clinics may impose rules that require patients to participate in counseling to receive buprenorphine prescriptions or discharge patients who use other substances, such as cocaine or cannabis, which also serves to stigmatize and exclude some patients [25]. At the individual level, stigma may deter some patients from seeking buprenorphine treatment due to fears of being judged or labeled as an “addict” [5]. Healthcare providers’ misconceptions about medications for OUD (MOUD), concerns about diversion, and discomfort managing OUD are associated lower willingness to prescribe buprenorphine [26–29]. Providers holding negative perception and prejudicial views may also manifest in demeaning communication and biased documentation affecting quality of care [30].

Innovative “low-threshold” models of buprenorphine treatment, such as co-locating prescribers at syringe services programs (SSPs), show promise in overcoming barriers that people with OUD face in part by offering treatment in settings where substance use is less stigmatized [31–34]. The principles of low-threshold buprenorphine treatment, which include flexible patient-centered policies and procedures and a harm reduction orientation (e.g., accepting potential goals like reducing opioid use as opposed to achieving abstinence), emphasize increasing access to care and patient-centeredness [35]. Low-threshold models may also reduce the stigma patients experience while engaging in OUD treatment [31]. However, implementing low-threshold buprenorphine treatment at SSPs can be challenging, including difficulty hiring buprenorphine providers comfortable with harm reduction values and practices [36]. Some SSP clients with OUD choose to use non-prescribed buprenorphine due to difficulties engaging in formal treatment, and concerns about being stigmatized by prescribers may be an important barrier [37, 38].

In this cross-sectional study, we used data from a randomized controlled trial of low-threshold buprenorphine treatment initiation at SSPs to investigate: (a) participants’ past experiences with substance use stigma, and (b) whether self-reported stigma was associated with buprenorphine treatment uptake in the trial. We hypothesized that participants reporting higher (vs. lower) levels of substance use stigma would have less success engaging in buprenorphine treatment. The findings from this study could help explain the impact of stigma on treatment engagement, ultimately contributing to more effective and patient-centered care.

## Methods

The parent study was conducted at three SSPs in New York City between 2020 and 2024. Participants, who were SSP clients, provided written informed consent, completed questionnaires at baseline, including a validated substance use stigma instrument, and were randomized to receive onsite buprenorphine treatment initiation or referral to a federally qualified health center (FQHC) for buprenorphine treatment [39]. This secondary analysis used data collected at baseline and the one-month follow-up visit. The study was approved by the Albert Einstein College of Medicine Institutional Review Board.

### Setting

The three SSPs had prior experience with clinical research. Two of the SSPs participated in research that informed development of onsite treatment protocols [40]. SSP sites served low-income neighborhoods with mostly Black and Latinx populations and among the highest opioid overdose mortality rates in New York City. An FQHC within the Montefiore Health System, which had previously worked with the SSPs, was the site for the referral condition.

### Onsite Buprenorphine Treatment Initiation

The parent clinical trial tested a two-week bridging intervention in which a trained study clinician evaluated participants for buprenorphine treatment at the SSPs, saw participants twice for weekly visits and buprenorphine prescriptions, and then made a referral for long-term buprenorphine treatment to the same FQHC used for the referral condition. Thus, the intervention expedited buprenorphine treatment initiation, but participants randomized to the intervention arm were still expected to follow-up at the FQHC following the two-week bridging intervention.

### Participants

Eligibility criteria were: age ≥ 18 years old, OUD by DSM-5 criteria (assessed by a trained research coordinator) [41], interest in and motivation for buprenorphine treatment, absence of contraindications to buprenorphine, and willingness to be randomized (i.e., accept a referral to the FQHC). Pregnancy or untreated mental illness were exclusions.

### Data collection

Participants completed study visits either in-person or remotely at the time of their enrollment (baseline) and at 2, 4, 8, 12, and 24 weeks post-enrollment. Study visits included completing computerized questionnaires and providing a urine sample for toxicology testing (if visits were conducted in-person). Study data were collected and managed using REDCap (Research Electronic Data Capture), a secure, web-based electronic data capture platform hosted at Albert Einstein College of Medicine [42, 43]. Participants were compensated for their study visits. All data presented here were from the baseline study visit, except for the main outcome variable, engagement in buprenorphine treatment, which was assessed at the 4-week study visit.

### Key variables

*Primary outcome* - The primary outcome for the clinical trial and this secondary analysis was buprenorphine engagement, defined as having an active buprenorphine prescription at 30 days (+/- 3 days), operationalized by recording the dates of buprenorphine prescriptions and number of days of buprenorphine supplied, then counting forward from the prescription date to determine whether the participant would have been expected to have buprenorphine on at least one day during the period 27–33 days post-enrollment. This outcome was modeled from clinical trials that tested buprenorphine treatment initiation in other novel settings (i.e., emergency department and inpatient medical wards) and then examined successful linkage to ongoing buprenorphine treatment [44, 45]. Because the onsite buprenorphine treatment intervention at SSPs only included 2-weeks of buprenorphine, the 30-day outcome represented successful linkage for long-term buprenorphine treatment [39].

*Substance Use Stigma* - The Ahern stigma scale was used to assess stigma related to substance use [7]. Items of this scale have been adapted from previously validated stigma scales and reframed for illegal drug use contexts [46–48]. Its reliability has been demonstrated through assessments of internal consistency and construct validity, supporting its use in measuring drug use-related stigma and its associations with relevant health and social outcomes. There are 15 items across four domains (discrimination, alienation, perceived devaluation, coping response) where respondents endorse whether they have ever had these experiences (dichotomous, yes/no). Summary scores for each domain were calculated by summing the number of affirmative responses to items in each domain. An individual item, “Have you been prevented from obtaining medical care because you use drugs?” was also analyzed individually because of the direct relevance to buprenorphine treatment engagement.

### Covariates

Demographic characteristics included age, gender, race/ethnicity, and housing stability. Clinical covariates included the severity of OUD (number of DSM-5 criteria met) [41], substance use in the prior 30 days, injection drug use in the prior 30 days (dichotomous, yes/no), mental health comorbidity (assessed via self-report individually, reported here as any of the following: depression, anxiety, bipolar disorder, schizophrenia, or posttraumatic stress disorder (PTSD)), and any prior buprenorphine treatment (dichotomous, yes/no). Because buprenorphine engagement was not significantly different in the two study arms, the randomization assignment was not used as a covariate.

### Analysis

We used descriptive statistics to summarize participants’ characteristics based on whether they successfully engaged in buprenorphine treatment. Specifically, we report means and standard deviations for normally distributed continuous variables, medians and interquartile ranges (IQR) for skewed continuous variables, and frequencies and percentages for categorical variables. We operationalized perceived stigma in two ways: a summary score across three domains (discrimination, alienation, perceived devaluation) and individual domain scores. The total summary score included 10 items with a higher score representing greater perceived stigma. The discrimination domain included 4 items (scored 0–4), the alienation domain included 3 items (scored 0–3), and the perceived devaluation domain included 3 items (scored 0–3). We constructed separate multivariable logistic regression models with operationalized stigma variables as the main independent variables and buprenorphine engagement as the dependent variable. Different models used, respectively, the total stigma score, discrimination domain, alienation domain, perceived devaluation domain, and discrimination in medical care item as the main independent variable. Covariates, including injection drug use (past 30 day), unstable housing, mental health comorbidity, and prior buprenorphine treatment, were chosen for the regression models a priori based on clinical relevance for buprenorphine engagement. This approach explores perceived stigma as a predictor of buprenorphine engagement among all participants regardless of study arm.

## Results

The study sample consisted of 97 participants with OUD, with a mean age of 42 years (SD ± 10.6), the majority (78.4%) being male. Racial and ethnic composition included 53.6% Hispanic, 29.9% Non-Hispanic Black, and 16.5% Non-Hispanic White. Participants commonly reported past-30 day injection drug use (62.9%) and near-daily heroin use (70.1%). Almost half (45.4%) had a self-reportedmental health condition such as depression, bipolar disorder, anxiety, psychotic disorders, or PTSD. None of these characteristics were significantly associated with buprenorphine engagement at 30 days (see Table 1).

**Table 1:** Baseline characteristics for syringe services program participants with opioid use disorder (OUD) by buprenorphine engagement (*N* = 97)

Participant characteristic, *n* (%)	Total (*n* = 97)	Not engaged (*n* = 58)	Engaged (*n* = 39)	*P*-value
Age, mean (SD)	42.0 (± 10.6)	42.1 (± 10.7)	41.9 (± 10.6)	NS
Male	76 (78.4)	46 (79.3)	30 (76.9)	NS
Race/Ethnicity				NS
Non-Hispanic Black	29 (29.9)	16 (27.6)	13 (33.3)	
Non-Hispanic White	16 (16.5)	13 (22.4)	3 (7.7)	
Hispanic	52 (53.6)	29 (50.0)	23 (59.0)	
Unstable Housing^a^	42 (43.3)	28 (48.3)	14 (35.9)	NS
Clinical				
Any Mental Health Condition^b^	44 (45.4)	27 (46.6)	17 (43.6)	NS
Intravenous Drug use, any^c^	61 (62.9)	40 (69.0)	21 (53.9)	NS
Prior buprenorphine treatment	18 (18.6)	10 (17.2)	8 (20.5)	NS
OUD criteria^d^, mean (SD)	10.6 (± 2.1)	10.5 (± 2.1)	10.9 (± 2.2)	NS
OUD severe^e^	94 (96.9)	56 (96.6)	38 (97.4)	NS
Near daily heroin use^f^	68 (70.1)	45 (77.6)	16 (41.0)	0.05
Methadone, any^c^	13 (13.4)	6 (10.3)	7 (18.0)	NS
Alcohol, any^c^	31 (32.0)	18 (31.0)	13 (33.3)	NS
Near daily cocaine use^e^	20 (20.6)	14 (24.1)	6 (15.4)	NS

^a^unstable housing = homeless or living in shelter | ^b^ depression, bipolar disorder, anxiety disorder, psychotic disorder, or PTSD | ^c^ past 30 days | ^d^ total number of DSM-5 criteria for opioid use disorder | ^e^ OUD severe = ≥ 6 DSM-5 criteria | ^f^ near daily = self-reported use on ≥ 27 days out of the previous 30

**Fig. 1:**
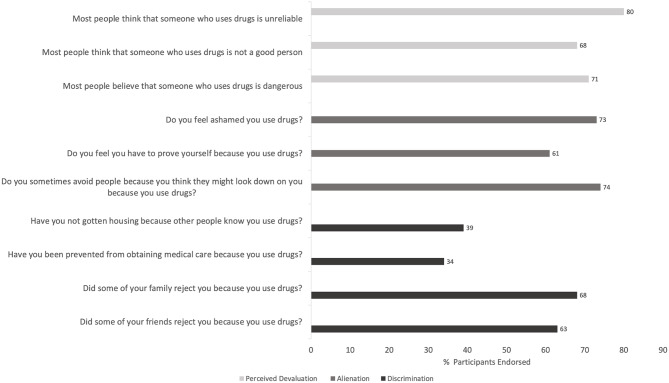

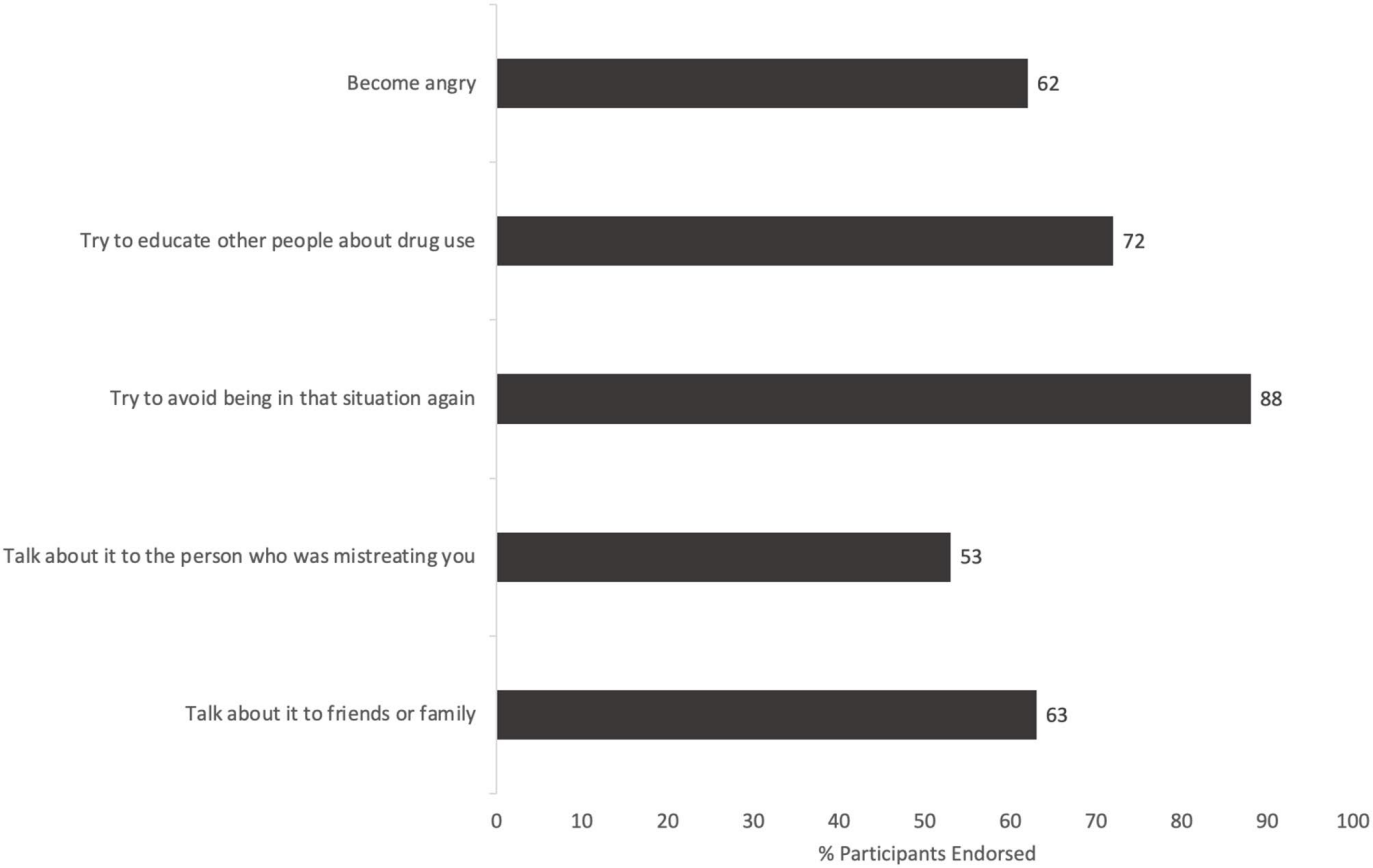
**a**. Perceived substance use stigma among syringe services program participants interested in buprenorphine treatment (*N* = 97). **b**. Self-reported coping strategies for substance use stigma among syringe services program participants (*N* = 97). Participants reported stigma across four domains of the Ahern substance use stigma scale: discrimination, alienation, perceived devaluation, and coping strategies [7]. Bars represent the proportion of participants endorsing at least one item within each domain

Participants reported high levels of stigma across all domains, as shown in Fig. 1. Over 60% experienced stigma in each category, except for the items describing being prevented from receiving medical care or denied housing due to substance use. Specifically, 76 of 97 participants (78.3%) endorsed at least one item in the discrimination domain, 82 of 96 (85.4%) reported at least one item in the alienation domain, and 81 of 96 (84.4%) reported at least one item in the perceived devaluation domain. When total scores were tallied, the median number of endorsed items was 7 (IQR = 4.5 to 9).

**Fig. 2:**
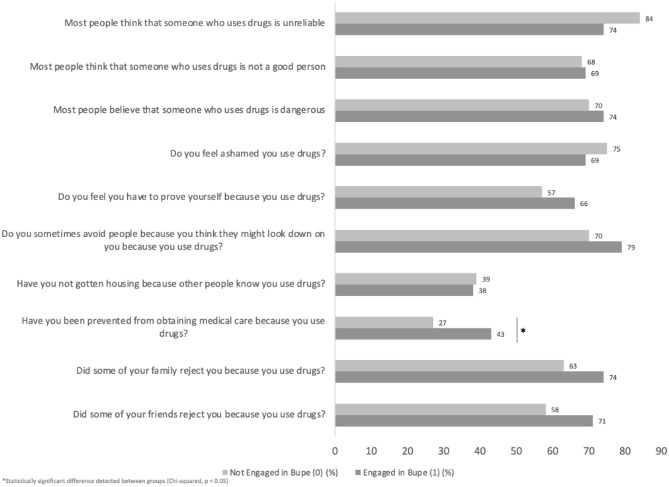
Differences based on buprenorphine engagement in perceived substance use stigma among syringe services program participants (*N* = 97). Bars represent the proportion of participants endorsing each item stratified by buprenorphine engagement status

When comparing participants who did and did not engage in buprenorphine treatment at 30 days, there were no significant differences in the overall stigma summary score, or any of the four domains [Figure 2] in unadjusted analyses. As for individual items, participants engaged in buprenorphine treatment at 30 days were significantly more likely to report having been prevented from receiving medical care due to substance use, when compared to those who were not engaged. This relationship is shown in Fig. 2.

In multivariable analysis, including prior injection drug use, unstable housing, mental health comorbidity, and prior buprenorphine treatment, the overall substance use stigma summary score was not significantly associated with 30-day buprenorphine engagement (see Table 2). The item on discrimination in medical care (see Table 2) was significantly associated with 30-day buprenorphine treatment engagement in the multivariable model (adjusted odds ratio: 2.64, 95% confidence interval: 1.05–6.66).

**Table 2:** Multivariable regression model examining the association between experiencing discrimination in medical care and buprenorphine engagement (*N* = 97)

Variable	Odds ratio	95% confidence internal	*p*-value
Denied medical care^a^	2.64	1.05–6.66	0.04
Injection drug use^b^	0.49	0.19–1.21	0.12
Unstable housing^c^	0.65	0.26–1.59	0.35
Mental illness^d^	0.93	0.39–2.22	0.87
Any prior buprenorphine	0.63	0.25–1.59	0.33

^a^ Denied medical care = self-reported experience of being denied care by a healthcare provider due to substance use |^b^ any injection drug use within the past 30 days | ^c^ report being homeless or living in a shelter | ^d^ Mental illness = depression, bipolar disorder, anxiety disorder, psychotic disorder, or PTSD |

## Discussion

This analysis demonstrates that most participants in this randomized control trial of low-threshold buprenorphine treatment at SSPs reported stigma in each domain of the Ahern substance use stigma scale reflecting high levels of perceived stigma. Overall, the total stigma score was not associated with buprenorphine treatment engagement at 30 days, but participants who had (vs. had not) previously been denied medical care due to substance use were more likely to initiate and continue buprenorphine treatment. This contradicts our hypothesis which proposed that participants with higher levels of substance use stigma would be less likely to engage in treatment.

Other studies have yielded findings different to those in this study regarding the relationship between perceived stigma and access to medical care. In a qualitative study, participants taking methadone or buprenorphine reported delays in seeking care, prematurely discontinuing medications, and forgoing other recovery activities because of perceived stigma related to SUDs [49]. Additionally, a study conducted among people who use drugs in need of hepatitis C treatment found that individuals with higher enacted stigma sought hepatitis C treatment less frequently, and those with greater internalized and enacted stigma were less likely to openly communicate with medical providers [15]. More broadly within the mental health stigma literature, a study examining 1660 adults with mental illness found that 28% of participants reported challenges in accessing medical care and 13% of participants attributed this to stigma [50]. Similarly, research with low-income public insurance beneficiaries found that stigma surrounding public insurance coverage led to adverse interactions (e.g. shaming, mistreatment, being disrespected or ignored) in healthcare [51]. These findings are different from our study where most stigma measures were not associated with buprenorphine engagement.

We hypothesized that stigma would be a barrier to buprenorphine engagement, but this relationship was not supported by our data. Participants who engaged in buprenorphine treatment more commonly reported having been previously prevented from receiving medical care due to substance use, but this question may be identifying a group that frequently seeks care, and therefore has more opportunities to experience stigma or discrimination, or a particularly resilient subgroup that is able to engage in needed medical care despite being denied care previously. The Ahern questionnaire did not directly assess whether participants were mistreated in healthcare settings or prior SUD treatment, which has been widely reported in research involving people with SUDs [17, 18]. Future research examining stigma and access to medical care should more directly measure whether participants anticipate that they will be stigmatized or receive substandard care due to their substance use. A different stigma measure, the Substance Use Stigma Mechanisms Scale, differentiates between perceived, anticipated, and internalized stigma and may be a better instrument for this type of analysis [52]. In the parent study, we also conducted qualitative interviews with participants, so we will be able to report whether stigma played a role in whether they engaged in buprenorphine treatment during the trial. It is plausible that the low-threshold approach to buprenorphine treatment offered in this trial was particularly effective for those who had been previously denied medical care due to substance use, but this study design does not allow for strong causal inference.

The finding that most participants perceived high levels of substance use stigma emphasizes the importance of low-barrier non-judgmental services when trying to engage SSP participants or other marginalized people who use drugs. Stigma was prevalent among most participants in the trial underscoring that people who engage in SUD treatment carry these negative experiences and attitudes with them. Most participants experienced discrimination and reported that their friends and family rejected them due to their substance use. Additionally, most participants experienced alienation feeling that they must prove themselves because of their substance use or that they are looked down upon due to drugs. Most participants believed that, in general, people see drug users as dangerous and unreliable. These perceptions should be acknowledged in all healthcare settings, and quality improvement initiatives could focus on making medical care, including OUD treatment, more welcoming to people who use drugs.

There are several potential ways to reduce SUD-related stigma in healthcare settings. Emerging evidence does support provider-focused stigma interventions. Educational and interaction-based interventions for healthcare providers are associated with significant reductions in stigmatizing attitudes toward people with SUDs [53, 54]. There is likely also a role for interprofessional harm reduction trainings that integrate experiential and practice-based components [55]. Addressing provider misconceptions about MOUD could also counteract a reported barrier to buprenorphine prescribing [27, 28], and learning to prescribe buprenorphine has been associated with improved attitudes toward SUDs among healthcare providers [56]. Reducing societal stigma toward SUDs would require broader action though.

This study has several limitations that should be considered. First, regarding generalizability, data were from a clinical trial in a single urban area with modest sample size, so participants may not be representative of other geographic areas or the broader population of people with OUD. The sample was predominantly people of color, and the influence of other identities or the role of intersectionality, was not examined. People from different racial/ethnic groups may report stigma toward OUD differently [57], which deserves attention in future research. Second, as mentioned above, our measure of being denied medical care did not differentiate between experienced and anticipated stigma. Third, the follow up period was limited to 30 days, which may not capture the impact of stigma on long term retention in buprenorphine treatment. Fourth, the study did not specifically examine the impact of fentanyl, which was commonly present in non-prescribed opioid supplies in New York City during the study period. Fentanyl likely impacted engagement rates, but we cannot be sure whether it played a role in participants’ perception of stigma. Fifth, we did not collect data on how many times participants had sought SUD treatment, which could have helped contextualize the findings. Finally, we were unable to explore the specific reasons why some participants did not continue to engage in care; however future analyses of the qualitative interviews collected from participants could provide insights.

Overall, the findings highlight the intensity of stigma reported by SSP participants seeking buprenorphine treatment and indicate a need to reduce stigma toward people with SUDs in healthcare settings, including OUD care. Low-threshold buprenorphine treatment programs may be well-suited for people who have experienced SUD-related stigma in medical care, but additional research is needed to evaluate these innovative models of care. Nonetheless, the high prevalence of perceived stigma among participants underscores the need for broader interventions within healthcare systems serving people with SUDs. Further research could investigate whether reducing stigma increases engagement in SUD treatment but delivering affirming and patient-centered care should be a universal standard now.

## Data Availability

The data that support the findings of this study are available from the senior author upon reasonable request.

## Abbreviations

DSM-5: Diagnostic and Statistical Manual, 5th edition
IQR: Interquartile Range
OUD: Opioid Use Disorder
PTSD: Post-traumatic Stress Disorder
PWID: People Who Inject Drugs
SUD: Substance Use Disorder
